# Supplementary figures and images for: Cocoa, livelihoods, and deforestation within the Tridom landscape in the Congo Basin: A spatial analysis
Source: PLoS One. 2024 Jun 13;19(6):e0302598. doi: 10.1371/journal.pone.0302598 (PMC11175426; doi:10.1371/journal.pone.0302598)

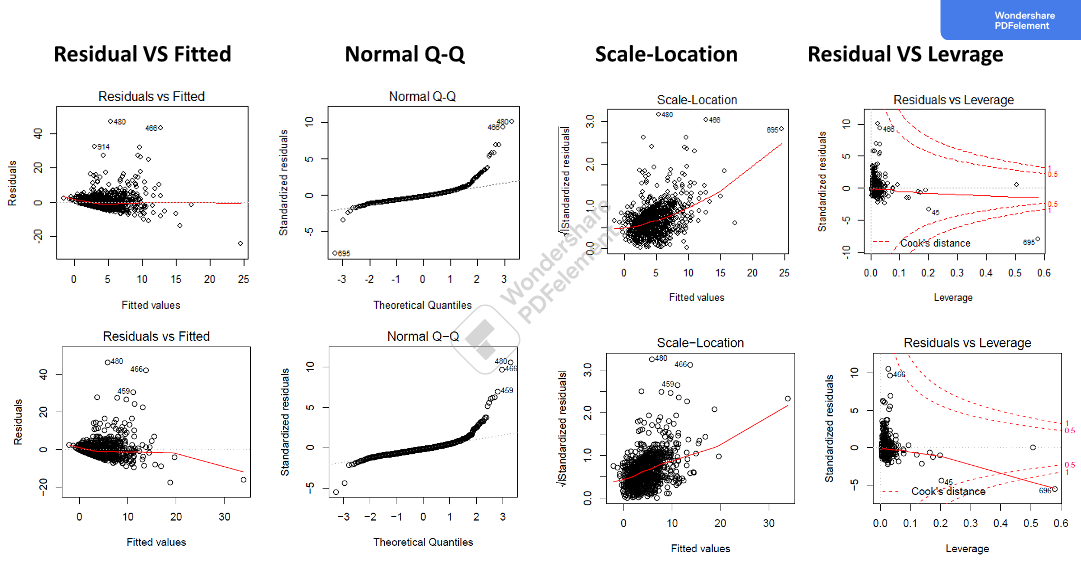

Supplement: S1 Fig — Diagnostic Plots for Regression Analysis. (TIFF) [file pone.0302598.s001.tiff]

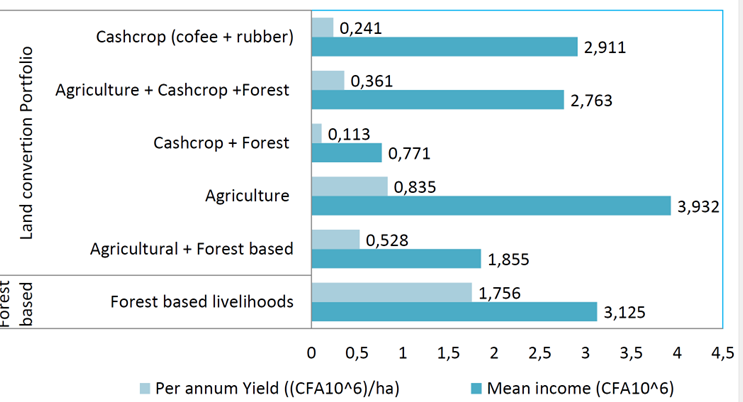

Supplement: S2 Fig — Livelihood Strategies and Per Annum Yiels/ha. (TIF) [file pone.0302598.s002.tif]

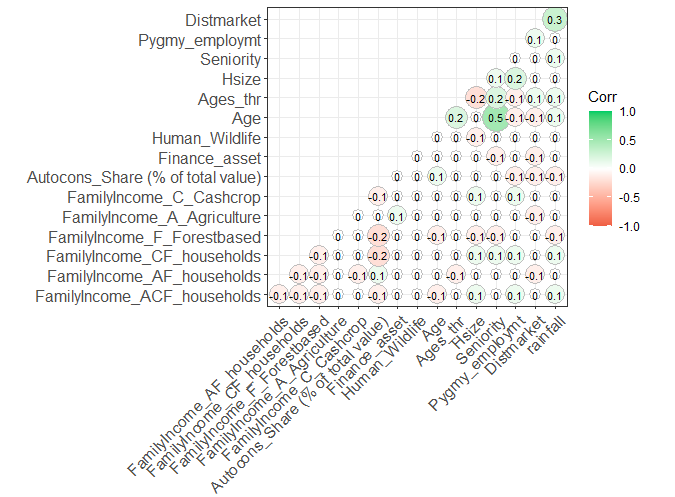

Supplement: S3 Fig — To assess the high correlation and to detect the possible presence of multicollinearity in the data, the study applied the Pearson correlation matrix. Results of Pearson’s correlation matrix indicated that the highest correlation among variables was 0.5; hence, there is no issue of multicollinearity. (TIF) [file pone.0302598.s003.tif]

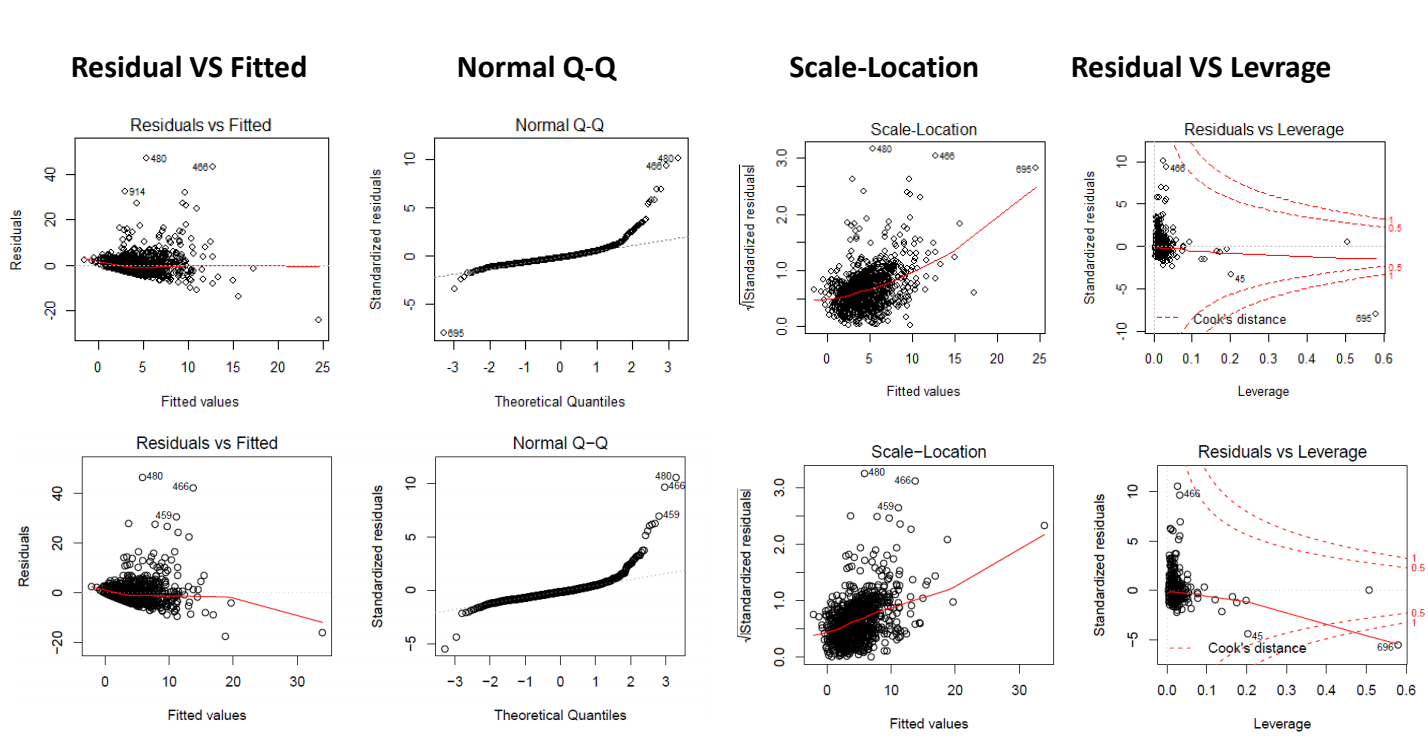

Supplement: S1 Appendix — (TIFF) [file pone.0302598.s007.tiff]

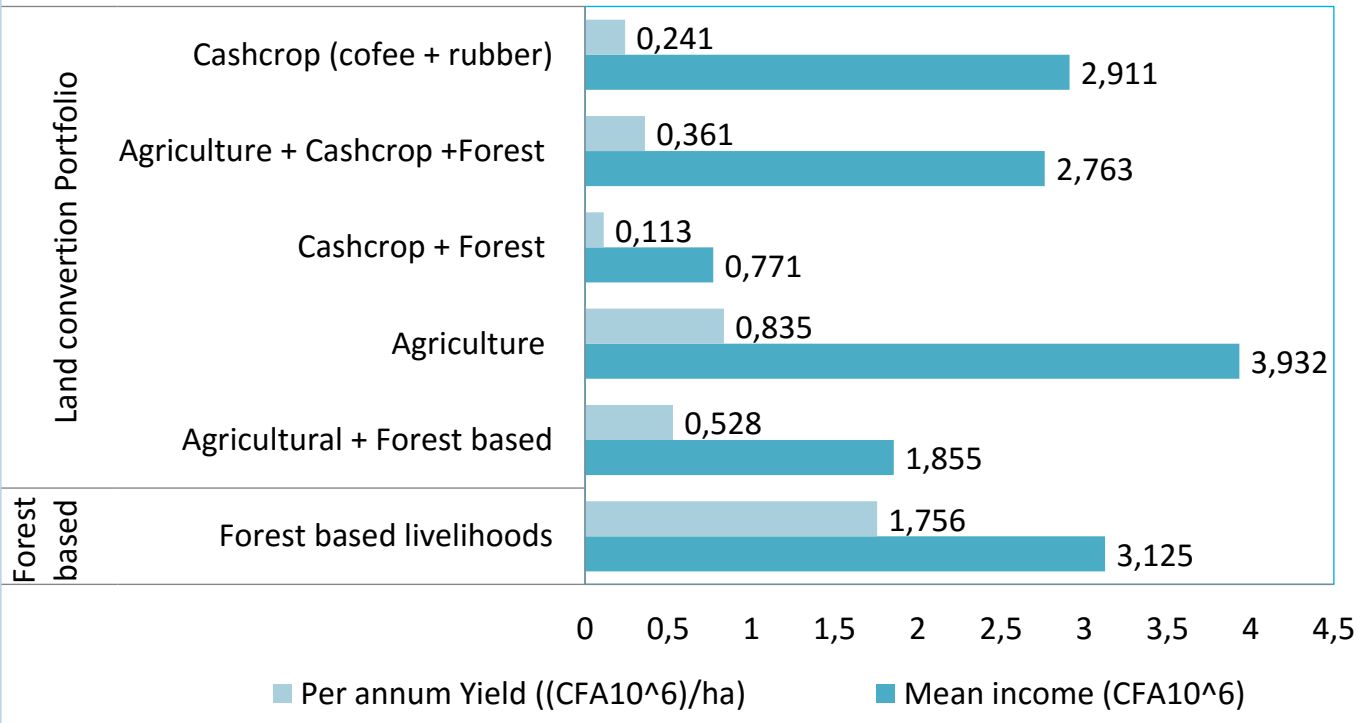

Supplement: S2 Appendix — (TIFF) [file pone.0302598.s008.tiff]
