# Supplementary material for: Cocoa, livelihoods, and deforestation within the Tridom landscape in the Congo Basin: A spatial analysis
Source: PLoS One. 2024 Jun 13;19(6):e0302598. doi: 10.1371/journal.pone.0302598 (PMC11175426; doi:10.1371/journal.pone.0302598)
Supplement: S1 Table — Full Spatial Autoregressive Model with all the explanatory variables. (ZIP) [file pone.0302598.s004.zip › S1_Table.pdf]

S1 table. Full Model. Full Spatial Autoregressive Model with all the explanatory variables.

	SPATIAL AUTOREGRESSIVE FULL MODEL				
	Coef.	SD	z value	Pr(>—z—)	
(Intercept)	-4,1857	2,2804	-1,8355	0,0664	*
**AGENT'S DECISION PARAMETERS**					
**Income from diversification strategies (FCFA10^3)**					
ACF_households	0,0101	0,0013	7,905	0	***
AF_households	0,0024	0,0012	2,0399	0,0414	**
CF_CocoaForest	0,0109	0,0021	5,326	0	***
**Income from specialization strategies (FCFA10^3)**					
F_Forestbased	-0,0002	0,0004	-0,4287	0,6682	
A_Agriculture	0,0026	0,0012	2,2638	0,0236	**
C_Cocoa	0,0178	0,0021	8,3798	0	***
**Other decision variables**					
Autocons_Share (% of total value)	-2,2003	0,7772	-2,8308	0,0046	***
**Capital & factor constraints**					
Finance_asset (FCFA10^3)	0,0089	0,0043	2,0988	0,0358	**
Landconflict Dummy (1=yes)	0,33	0,3763	0,8771	0,3804	
Human_Wildlife (FCFA10^3)	-0,2033	0,0978	-2,0785	0,0377	**
**HOUSEHOLDS CHARACTERISTICS**					
Gender (1=Male)	0,6824	0,3508	1,9451	0,0518	*
Age (continuous. in years)	0,0208	0,0121	1,7115	0,087	*
Ages_thr	-0,0014	0,0006	-2,2333	0,0255	**
Marit_single (1=Maried)	0,42	0,3485	1,2051	0,2282	
Hsize (continuous)	0,1582	0,0376	4,2037	0	***
Schoolcycl_2 (1=secondary school)	-0,1703	0,314	-0,5423	0,5876	
Autochbaka (1=Baka. 0=Bantou)	-0,5574	0,6761	-0,8244	0,4097	
Seniority (continuous. in years)	0,0391	0,008	4,859	0	***
CommunityGroup Dummy (1=yes)	0,524	0,3235	1,62	0,1052	
Baka_employement (coutinuous)	0,1518	0,0504	3,0118	0,0026	***
laborduration	-0,0483	0,0319	-1,5107	0,1309	
**CONTEXTUAL VARIABLES**					
Country (1=Cameroun. 0=Gabon)	0,8112	0,4593	1,7661	0,0774	*
Distmarket Km	-0,0037	0,0029	-1,2952	0,1952	
Distance to P. Areas (Km)	0,0006	0,0066	0,0903	0,928	
**Biophysical factor**					
Rainfall	0,0014	0,0014	0,9974	0,3186	
Rho (ρ)	0.1947	**			
Log Likelihood	-2849				
ML residual σ	4.350				
AIC Criterion	5754				
Wald Statistic	4.8115	**			
Observations	986				
residual autocorrelation	0.0728				
*, ** and *** = significance level at 1% 5% 10% respectively					
