# Supplementary material for: Cocoa, livelihoods, and deforestation within the Tridom landscape in the Congo Basin: A spatial analysis
Source: PLoS One. 2024 Jun 13;19(6):e0302598. doi: 10.1371/journal.pone.0302598 (PMC11175426; doi:10.1371/journal.pone.0302598)
Supplement: S2 Table — Estimations with Instrumental variables method to control for endogeneity bias. (ZIP) [file pone.0302598.s005.zip › S2_Table.pdf]

S2 table Instrumental variable. Estimations with Instrumental variables method to control for endogeneity bias.

	Deforest		
Souspicious variables	ACF_households (StD)	CF_households (StD)	C_Cocoa (StD)
ACF_households	0.0169***		
	(0.0046)		
AF_households	0.0020	-0.0008	-0.0009
	(0.0013)	(0.0017)	(0.0019)
CF_households	-0.0110		
	(0.0186)		
A_Agriculture	0.0025**	0.0016	0.0015
	(0.0013)	(0.0014)	(0.0016)
C_Cocoa	-0.0244		
	(0.0425)		
Autocons_Share	-3.4298***	-4.3714***	-4.7979**
	(0.8164)	(1.3105)	(2.0029)
Finance_asset	0.000001**	0.000001**	0.000001**
	(0.000000)	(0.000000)	(0.000000)
Human_Wildlife	-0.2215**	-0.2607**	-0.2633**
	(0.1051)	(0.1095)	(0.1206)
Gender	0.8354**	1.2092***	1.2315**
	(0.3718)	(0.4565)	(0.5130)
Age	0.0373***	0.0305**	0.0373**
	(0.0124)	(0.0127)	(0.0185)
Ages_thr	-0.0015**	-0.0017**	-0.0018**
	(0.0007)	(0.0007)	(0.0008)
Hsize	0.1852***	0.2498***	0.2646***
	(0.0426)	(0.0466)	(0.0658)
Seniority	0.0429***	0.0500***	0.0470***
	(0.0085)	(0.0105)	(0.0097)
CommunityGroup	0.4854	0.5559	0.3518
	(0.3488)	(0.3705)	(0.4789)
Pygmy_employmt	0.1959***	0.2039***	0.2280**
	(0.0541)	(0.0629)	(0.0944)
Country	1.1241***	1.9181***	1.5841***
	(0.4150)	(0.4579)	(0.5401)
Constant	-1.1159	-0.9463	-0.9219
	(0.7707)	(0.7961)	(0.8745)
Observations	986	986	986
Diagnostic tests:			
Weak instruments	17.525***	19.069***	4.283**
Wu-Hausman	2.700	1.464	1.095
Sargan	4.717	2.491	2.029
Note:	*p<0.1;	**p<0.05;	***p<0.01
