# Supplementary material for: Cocoa, livelihoods, and deforestation within the Tridom landscape in the Congo Basin: A spatial analysis
Source: PLoS One. 2024 Jun 13;19(6):e0302598. doi: 10.1371/journal.pone.0302598 (PMC11175426; doi:10.1371/journal.pone.0302598)
Supplement: S3 Table — Akaike’s information criterion (AIC) and Bayes’ information criterion (BIC): Comparison tests for models. (ZIP) [file pone.0302598.s006.zip › S3_Table.pdf]

S3 table Robustness check. Akaike's information criterion (AIC) and Bayes' information criterion (BIC): Comparison tests for models.

Weight_matrice	Model	nobs	logLik	deviance	AIC	BIC
GabGraph	SAR	986	-2, 856.8090	18, 962.6500	5, 751.6180	5, 844.5980
GabGraph	SEM	986	-2, 856.9640	18, 970.3600	5, 751.9280	5, 844.9080
GabGraph	SDM	986	-2, 838.8900	18, 287.6900	5, 747.7800	5, 919.0580
5 NN	SAR	986	-2, 855.0530	18, 877.8500	5, 748.1060	5, 841.0860
5 NN	SEM	986	-2, 855.7030	18, 904.1300	5, 749.4070	5, 842.3860
5 NN	SDM	986	-2, 843.0860	18, 443.1600	5, 756.1720	5, 927.4500
10 NN	SAR	986	-2, 852.0740	18, 733.0700	5, 742.1480	5, 835.1270
10 NN	SEM	986	-2, 853.9110	18, 805.7000	5, 745.8230	5, 838.8020
10 NN	SDM	986	-2, 840.0390	18, 328.1600	5, 750.0780	5, 921.3560
distance based	SAR	986	-2, 830.2090	17, 893.9500	5, 730.4190	5, 901.6970
distance based	SEM	986	-2, 854.5520	18, 839.3100	5, 747.1040	5, 840.0830
distance based	SDM	986	-2, 830.2090	17, 893.9500	5, 730.4190	5, 901.6970
