# Supplementary material for: Cocoa, livelihoods, and deforestation within the Tridom landscape in the Congo Basin: A spatial analysis
Source: PLoS One. 2024 Jun 13;19(6):e0302598. doi: 10.1371/journal.pone.0302598 (PMC11175426; doi:10.1371/journal.pone.0302598)

Cocoa, livelihoods, and deforestation in the Congo Basin: A spatial analysis

PONE-D-23-09897

Response to editor

We would like to thank the editors for the edits requested to improve our submission PONE-D-23-09897. We did our best to address the text overlap with our previously published works, and we hope they will find our revision satisfactory, and in compliance with the PLOS ONE submission guidelines. Below is our detailed response.

1. **Regarding the first paper (Sonwa et al. 2014) in the following link below: https://www.researchgate.net/publication/263275480_Plant_diversity_management_in_cocoa_agroforestry_systems_in_West_and_Central_Africa-effects_of_markets_and_household_needs**

We have carefully compared our PONE-D-23-09897 manuscript with this paper, and we found three places to cite the paper. We have now appropriately provided references where needed. Please, see line 34, line 38, and line 747 in the revised manuscript. Here, reference number [21] refers to Sonwa et al. 2014.

2. **Regarding the second paper I lead, following the link below: https://www.sciencedirect.com/science/article/abs/pii/S092180091530238X?via%3Dihub**

We have carefully compared our PONE-D-23-09897 manuscript with this second published paper (Ngouhouo-poufoun et al, 2016). We effectively found significant text overlap between both papers. The overlapping text was mainly on the presentation of data, the study area, the sampling, and the administration mode at lines 427 to 446 in the previous version of the manuscript. We also found one additional place where our PONE-D-23-09897 manuscript was missing to refer to our published paper Ngouhouo-Poufoun et al, 2016. We finally found that the table in Appendix B.1. in our PONE-D-23-09897 manuscript was identical to the one in Appendix B.2. in our published paper (Ngouhouo-poufoun et al, 2016). Please find below our actions to address the issues with the second paper:

1. The overlapping text in 427 to 446 in our PONE-D-23-09897 manuscript was rewritten. It was rephrased and shortened. We then referred to our published paper (Ngouhouo-Poufoun et al) numbered [73], for a more detailed presentation of the duplicated information. The reformulation now stands in lines 427 to 435 in the revised manuscript instead of 427 to 446 in the version previously submitted.

2. We have also reformulated the text in lines 288 to 291 and we have cited the published

paper (Ngouhouo-Poufoun et al, 2016, or [73] in an appropriate manner).

3. Finally, we have removed the table in Appendix B.1.entitled “Stratified sampling and spatial representativeness” in the previous version of our PONE-D-23-09897 manuscript. As this information is identical to the table in Appendix B.2. in our published paper (Ngouhouo-poufoun et al, 2016), we then refer to the published paper for further information on the sampling.

Dear Editor,

We Hope we have addressed the issues raised and that our revision is thorough. Please we are open to addressing any other specific or general aspect we might have forgotten.

Thank you

Yours faithfully

Jonas Ngouhouo-Poufoun, Ph.D.

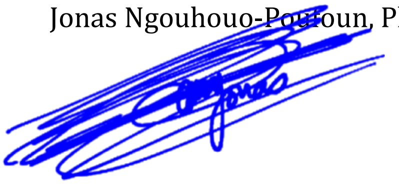

Supplement: Attachment — Submitted filename: Answer to Editors_PLONE.pdf [file pone.0302598.s009.pdf]
