# Supplementary material for: Cocoa, livelihoods, and deforestation within the Tridom landscape in the Congo Basin: A spatial analysis
Source: PLoS One. 2024 Jun 13;19(6):e0302598. doi: 10.1371/journal.pone.0302598 (PMC11175426; doi:10.1371/journal.pone.0302598)
Supplement: Attachment — Submitted filename: Review of Article.docx [file pone.0302598.s010.docx]

Review of Article

**Cocoa, livelihoods and deforestation in the Congo Basin: A spatial analysis**

By: Jonas Ngouhouo Poufoun, Sabine Chaupain-Guillot, Youba Ndiaye, Denis Sonwa, Kevin Njabo, Philippe Delacote.

Context:

This paper analyzes the full set of potential drivers of households’ deforestation, prioritizing or distinguishing among them to inform policy makers and facilitate appropriate political decision processes to curb deforestation from smallholders’ agriculture and forest activities in the medium- and long-term perspectives. Spatial autoregressive method was used for analysis of survey data from 1035 households in the Tridom landscape in the Congo basin. The result is that households emulate the deforestation decisions of their neighbors. Moreover, a marginal increase in income from cocoa-based livelihood’s portfolio, is associated with six to seven times higher deforestation than other livelihoods’ strategies with a significant spillover effect on neighboring households’ deforestation. Increased income from cocoa-based livelihoods in open access systems can negatively affect forests. This paper proposes as a policy recommendation that an approach to tackle the high risk of deforestation associated with cocoa could pass through farmers. This is done by promoting a complex cocoa agroforest system.

Comments:

It is a very valuable and relevant topic. It contributes significantly to the development of strategies for reducing deforestation associated with cocoa. The study presents original research results and has not been published elsewhere. Also, Authors have addressed the critical concerns raised by preview reviewers.

One point that I would like to raise is the use of a spatial autoregressive model (SAR) without prior justification. For example, why is a SAR model preferred to Spatial Durbin Model (SDM) or Spatial Error-correction Model (SEM)? Bayesian and AIC information criteria could be applied in order to confirm that SAR is the best method.
